# Effect of plano-valgus foot posture on midfoot kinematics during barefoot walking in an adolescent population

**DOI:** 10.1186/s13047-018-0297-7

**Published:** 2018-10-01

**Authors:** Paolo Caravaggi, Chiarella Sforza, Alberto Leardini, Nicola Portinaro, Artemisia Panou

**Affiliations:** 1 Movement Analysis Laboratory, IRCCS Istituto Ortopedico Rizzoli, Via di Barbiano 1/10, 40136 Bologna, Italy; 2 Department of Biomedical Sciences for Health, Università degli Studi di Milano, Via Mangiagalli, 31, 20133 Milan, Italy; 3 Department of Pediatric Orthopedics and Neuro-orthopedics, Humanitas Research Hospital, University of Milan, Via Manzoni 56, 20089 Rozzano, MI Italy; 4 Department of Translational Medicine, Orthopaedic and Traumatology Clinic, Humanitas Research Hospital, University of Milan, Rozzano, Milan Italy

**Keywords:** Flat-foot, Adolescents, Midfoot, Midtarsal joint, Tarso-metatarsal joint, Kinematics, Windlass mechanism, Medial longitudinal arch

## Abstract

**Background:**

Plano-valgus is a common alteration of the paediatric foot, characterized by valgus hindfoot, foot pronation and drop of the medial longitudinal arch. Despite their importance in the diagnosis and classification of plano-valgus foot condition, little information is available on functional alterations of the major joints spanning the medial longitudinal arch – i.e. midtarsal and tarso-metatarsal. Aim of the study was to provide objective description of the alterations in plano-valgus midfoot joints with respect to those in an age-matched normally-developed feet population.

**Methods:**

Twenty adolescents (13.3 ± 0.8 years) with bilateral plano-valgus feet underwent clinical examination and were gait-analysed via a validated 4-segment foot model. This allowed to measure static foot posture, kinematics of the main foot joints, and medial longitudinal arch deformation during walking at comfortable speed. Range of motion and temporal profiles of joint rotations were compared to those from a control population of age-matched adolescents with normally-developed feet.

**Results:**

The plano-valgus midtarsal joint was more dorsiflexed, everted and abducted than that in the control group, and showed reduced sagittal-plane RoM (plano-valgus = 15.9 degrees; control = 22.2 degrees; *P* <  0.01). The tarso-metarsal joint was more plantarflexed and adducted, and showed larger frontal-plane RoM. The MLA showed larger RoM and was lower throughout the stance phase of the gait cycle.

**Conclusion:**

Significant postural and kinematic alterations are present at the midtarsal and tarso-metarsal joints of adolescents with plano-valgus feet. Objective identification and quantification of plano-valgus foot alterations, via non-invasive gait-analysis, is relevant to improving the diagnosis of this condition and to evaluating the effect of conservative treatments and of surgical corrections by different techniques.

## Background

Plano-valgus foot (PV) is a complex 3D-deformity of the foot, often asymptomatic, characterized by plantarflexion and eversion of the calcaneus relative to the tibia, plantarflexion of the talus, dorsiflexion of the navicular and supination of the forefoot [1, 2]. The most obvious features characterizing PV are valgus hindfoot and flattening of the medial longitudinal arch (MLA) during weightbearing. The MLA starts developing at the age of 2 and becomes structurally mature at around 10–13 years old [2]. Accordingly, the incidence of PV changes with age: it is present in about 37–60% of 2–6 year old children, and in about 16–19% of adolescents - around 8–13 years old - after growth plates closure [3–5]. While morphological signs of flexible PV disappear in the unloaded condition, deformity is always present in rigid PV due to structural alterations, collagen and neuromuscular disorders [6, 7]. A Cochrane review has shown that the reported incidence of PV is limited by variable sampling, age groups and assessment measures, thus resulting in different findings [3]. It is generally recognized that PV signs decrease with age, and that joint hypermobility and body weight increase the incidence of PV at all ages.

Clinically, the paediatric PV midfoot is significantly collapsed in weightbearing. Therefore, it has been postulated that the PV midfoot is everted and dorsiflexed for longer during late stance, thus making push-off less efficient [8–11]. Later in life, extended hindfoot pronation and arch collapse may lead to anterior pelvic tilt, internal hip rotation, knee valgus and internal rotation of the tibia [12–15]. Since alterations of foot posture in static conditions are not always correlated to those occurring in dynamic conditions, gait analysis in combination with a number of multi-segment foot models has been increasingly used for objective quantification of the complex multi-planar kinematics characterizing PV. A review of the most relevant literature on this topic is reported in Table 1. Twomey et al. [15], using the Heidelberg foot measurement method, reported increased forefoot supination and greater MLA deformation during gait in children with low arched feet. Hosl et al. [13] reported increased hindfoot eversion with limited dorsiflexion and frontal range of motion (RoM), compensated by increased supination, abduction and sagittal RoM of the forefoot both in symptomatic and asymptomatic flat feet. Saraswat et al. [16] reported increased hindfoot eversion and plantar flexion along with increased midfoot dorsiflexion and pronation in PV. Kerr et al. [17] reported increased hindfoot eversion in static conditions, with compensating forefoot abduction and supination, in children with flat feet. Finally, Kothari et al. [18] reported increased hindfoot eversion compensated by forefoot supination.

**Table 1:** Review of the literature on functional evaluation of the pediatric PV during gait using multisegment foot models

Study	Model	population & age (years)	hindfoot/tibia	forefoot/hindfoot	MLA
Twomey et al. Gait & Posture, 2010 [15]	Heidelberg	*n* = 27 age = 11.2 ± 1.2		+supination	**+**drop
Hosl et al. Gait & Posture, 2014 [13]	Oxford Foot Model	*n* = 21 age = 11.0 ± 2.6	**-**dorsiflexion	**+**sagittal ROM	
			**+**eversion	**+**supination	
			**-**frontal ROM	**+**abduction	
Saraswat et al. Gait & Posture, 2014 [16]	Saraswat^a^	*n* = 10 age = 10.6 ± 1.6	**+**max eversion	**+**dorsiflexion	
			**+**plantarflexion	+pronation	
Kerr et al. Clin. Biomech., 2015 [17]	Oxford Foot Model	*n* = 29 age = 10.7 ± 3.5	**+**eversion	**+**abduction	
				**+**supination	
Kothari et al. Gait & Posture, 2015 [18]	Oxford Foot Model	*n* = 42 age = 11.9 ± 2.0	**+**eversion	**+**supination	

Hindfoot/tibia, forefoot/hindfoot and MLA (medial longitudinal arch) columns show significant increase (+) or decrease (−) in gait kinematic parameters with respect to the control group reported in the study. For each study, only asymptomatic flat-foot samples have been listed in the population column

^a^modified Shriners Hospitals for Greenville foot model

Despite the clinical relevance of midfoot posture, our current understanding of paediatric PV function during gait is related to hindfoot, forefoot and hallux motion. Since most of the clinical signs and postural alterations of PV concern a number of joints spanning the medial longitudinal arch, a thorough functional analysis of PV can not disregard the information on 3D static alignment and motion of the joints in the midfoot - i.e. midtarsal and tarso-metatarsal joints. Improving our understanding of midfoot joints alterations is critical to shed more light on the clinical signs and extent of PV deformity requiring intervention, and to assess the effectiveness of non-invasive approaches and of surgical treatments.

Therefore, in order to better characterize PV kinematics, a validated multisegment foot model comprising the midfoot segment was used in this study to assess differences in posture and gait between asymptomatic PV and healthy feet joints. It was hypothesized that static posture and gait kinematics of the PV midtarsal and tarso-metatarsal joints are significantly different from those in normal-arched healthy feet.

## Methods

### Participants

Twenty adolescents (13 M and 7 F; age: 13 ± 1 years; height: 164 ± 7 cm; weight: 53 ± 11 kg; shoe size: 38–44 EU) were recruited for the study following their diagnosis of bilateral asymptomatic PV by two paediatric orthopaedic surgeons (NP and AP). Ten age-matched adolescents (4 M and 6 F; age: 13 ± 1 years; height: 156 ± 10 cm; weight: 48 ± 12 kg; shoe size: 37–44 EU) with normally-developed feet (ND) were used as control group.

According to the clinical protocol of the hosting Institution, participants who had calcaneal valgosity > 16° were included in the study. This was measured as the relative angle between neutral calcaneal stance position (NCSP) to resting calcaneal stance position (RCSP). Radiological indicators of PV condition, such as the calcaneal pitch, lateral talo-first metatarsal angle, and talo-navicular coverage, were measured using weightbearing X-rays. Valgosity of the calcaneus was measured with a goniometer. Exclusion criteria were the following: lower limb musculoskeletal disorders; concomitant systemic diseases; clinical signs of joint laxity, and major lower limb trauma. Children practicing sports at a competitive level were also excluded. The study was performed according to the ethical standards of the Declaration of Helsinki (1964) and its later amendments. Acknowledgement of the Hospital’s IRB was granted (protocol n° 7/17) and parents’ informed consent was obtained for all children recruited in the study.

In order to limit inter-observer variability [19], a single experienced operator performed clinical, radiographic and instrumental evaluation of all participants.

### Kinematic analysis

**Fig. 1:**
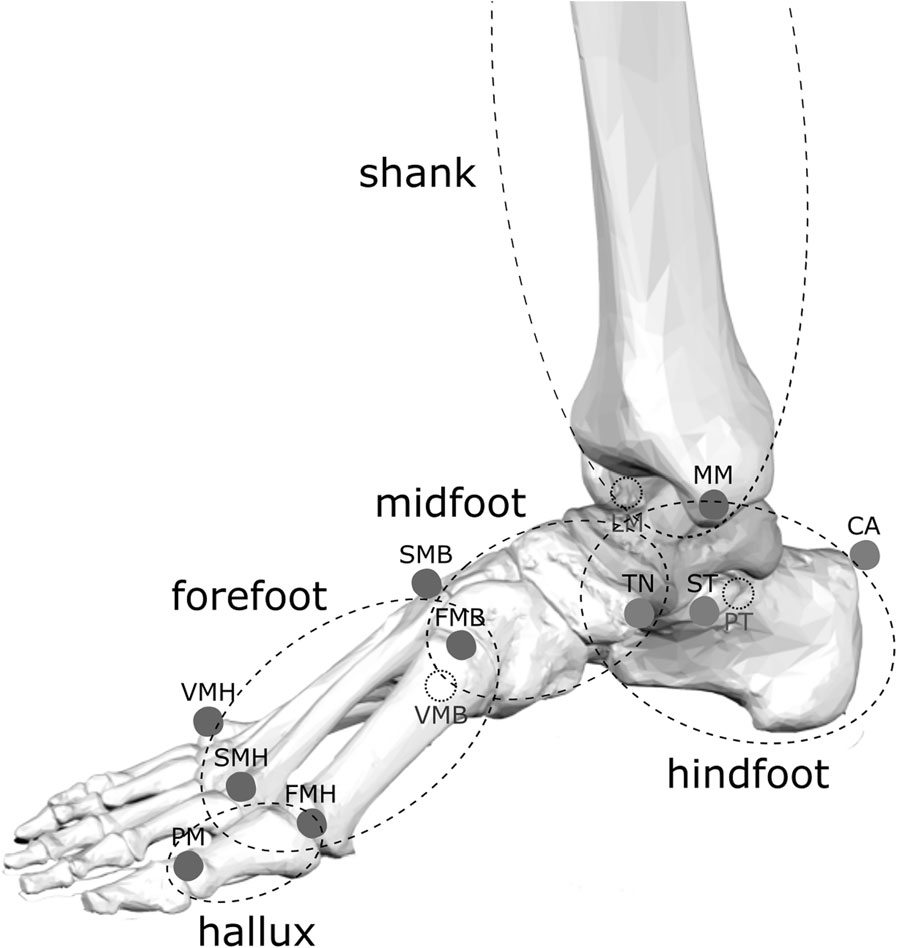
3D representation of the foot skeleton along with skin-markers attached to relevant bony landmarks according to the Rizzoli Foot Model [20, 32]. An anatomical reference frame is defined by using the markers’ position on each of the four segments: shank, hindfoot; midfoot; metatarsus, and hallux

**Fig. 2:**
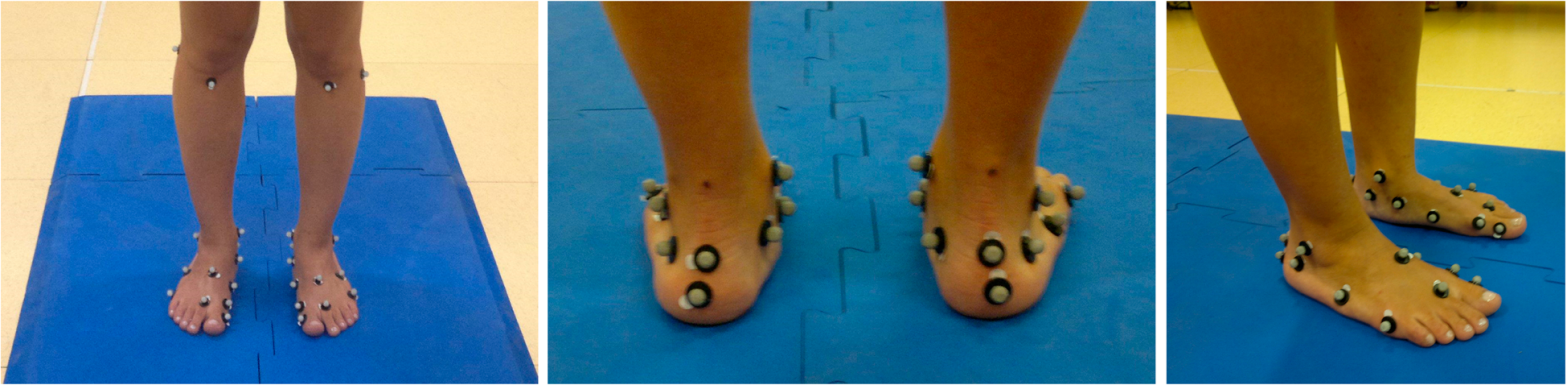
Left to right, front, rear and lateral view of the feet of one of the participants in the plano-valgus group. Sixteen reflective markers are attached to relevant bony landmarks according to the Rizzoli Foot Model for gait analysis

Sixteen 9 mm reflective spherical markers were applied via double-sided adhesive tape on anatomical landmarks on the left and right foot of each child, according to the Rizzoli Foot Model [20] (see Figs. 1 and 2). Foot markers trajectories were collected via a 6-camera motion capture system (Vicon Bonita B10, Vicon Motion System Ltd., Oxford, UK) sampling at 100 Hz. A double-leg support upright static posture was recorded for each participant. Before data acquisition, each participant was allowed to walk freely in the room for a few minutes to acclimatize with the marker set. A number of barefoot walking trials at comfortable speed were recorded for each participant. Following data pre-processing, three full gait cycles for each participant were used in the analysis. 3D joint rotations were calculated between: shank, i.e. tibia and fibula, and the combined foot segments; shank and calcaneus (i.e. ankle joint); calcaneus and midfoot (i.e. midtarsal joint); midfoot and metatarsus (i.e. tarso-metatarsal joint), and calcaneus and metatarsus (forefoot-to-hindfoot). Dorsiflexion/plantarflexion, abduction/adduction and eversion/inversion rotations were calculated at each joint in the sagittal, frontal and transverse plane respectively. Sagittal-plane rotation of the first metatarso-phalangeal joint, and deformation of the medial longitudinal arch (MLA) were also calculated. The Joint Coordinate System [21] was used to calculate joint rotations. Joint rotations were time-normalized to stride duration, which was determined from the analysis of the trajectories of the markers on the heel. RoM of each joint in the three anatomical planes was calculated as the absolute difference between the maximum and minimum angle recorded during stride duration.

### Statistics

Average left and right foot kinematic data were calculated across three walking trials for each participant in the PV and ND group, for a total of 60 samples. Mann-Whitney U test was used to assess differences in anthropometric (demographic) and kinematic parameters between PV and ND. A Bonferroni correction was applied to the significance level to account for the multiple comparisons in static posture and RoM between the two groups (α = 0.01). A post-hoc power analysis confirmed that the samples used for statistical analysis of midtarsal and tarso-metatarsal joints RoM in the three anatomical planes were sufficient to identify statistical differences between the two groups with a power of 0.8. One-dimensional statistical parametric mapping [22] was used to determine differences in time-histories of joint rotations between PV and ND.

## Results

No significant differences in age, height, and body mass were observed between groups. All PV radiological and clinical measurements were consistent with a diagnosis of flexible plano-valgus foot [23–26] and confirmed that feet in the PV group were clinically different from those in the ND group (Table 2). Normalized walking speed and stride length were lower in the PV group (Table 3).

**Table 2:** Clinical parameters in the plano-valgus and normally-developed feet populations

Clinical parameter	Plano Valgus	Normally developed
Talo-navicular coverage [deg]	24.2 (7.0)	< 7^a^
Meary’s angle [deg]	14.8 (5.1)	−4 - 4^a^
Calcaneal pitch [deg]	17.1 (6.1)	10–20^a^
Hindfoot valgosity [deg]	23.6 (4.5)	−5 – 5^a^

^a^Radiographic normative data for normally developed feet were extracted from [23, 24, 26]

**Table 3:** Mean (SD) spatio-temporal parameters in the plano-valgus and normally-developed feet populations

	Plano Valgus	Normally Developed	*p*
stance duration [% gait cycle]	59.6 (2.6)	59.1 (1.6)	NS
stride length [m]	1.14 (0.16)	1.29 (0.11)	< 0.001
stride length normalized [%h]	0.70 (0.06)	0.83 (0.05)	< 0.001
gait speed [m/s]	0.96 (0.14)	1.22 (0.15)	< 0.001
gait speed normalized [%h*s^−1^]	0.59 (0.06)	0.78 (0.08)	< 0.001

Mann-Whitney statistical significant differences between the two groups are reported in the last column. NS indicates non-statistically significative difference (Mann-Whitney, *p* >  0.05) between PV and ND

### Static posture

**Fig. 3:**
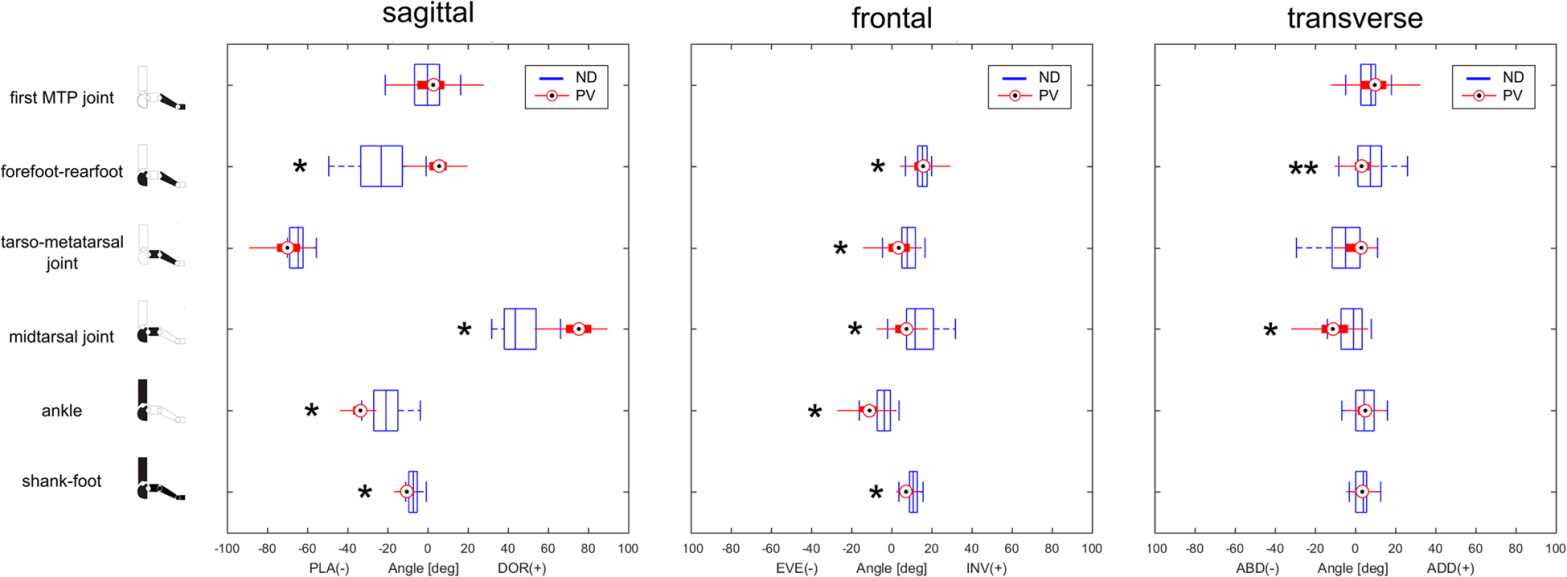
Left to right, distribution (median and 25–75 percentiles boxes) of the sagittal, frontal and transverse planes joint angles in the plano-valgus (PV) and normally-developed feet (ND) groups recorded during double-leg support upright static posture. * indicates statistically significant difference in joint angle between the two groups (Mann-Whitney test, α = 0.01)

Figure 3 shows the comparison of the foot joints positions during double-leg support static posture between PV and ND, respectively, in the sagittal, frontal and transverse plane. The calcaneus was more plantarflexed and everted with respect to the shank in the PV group. The midfoot was more dorsiflexed and everted with respect to the calcaneus. The metatarsus was more everted with respect to the midfoot and more dorsiflexed with respect to the calcaneus. In the transverse plane, the midfoot and metatarsus were more abducted relative to the calcaneus, although the latter was not statistically different (*P* >  0.01).

### Temporal profiles of joint rotations

**Fig. 4:**
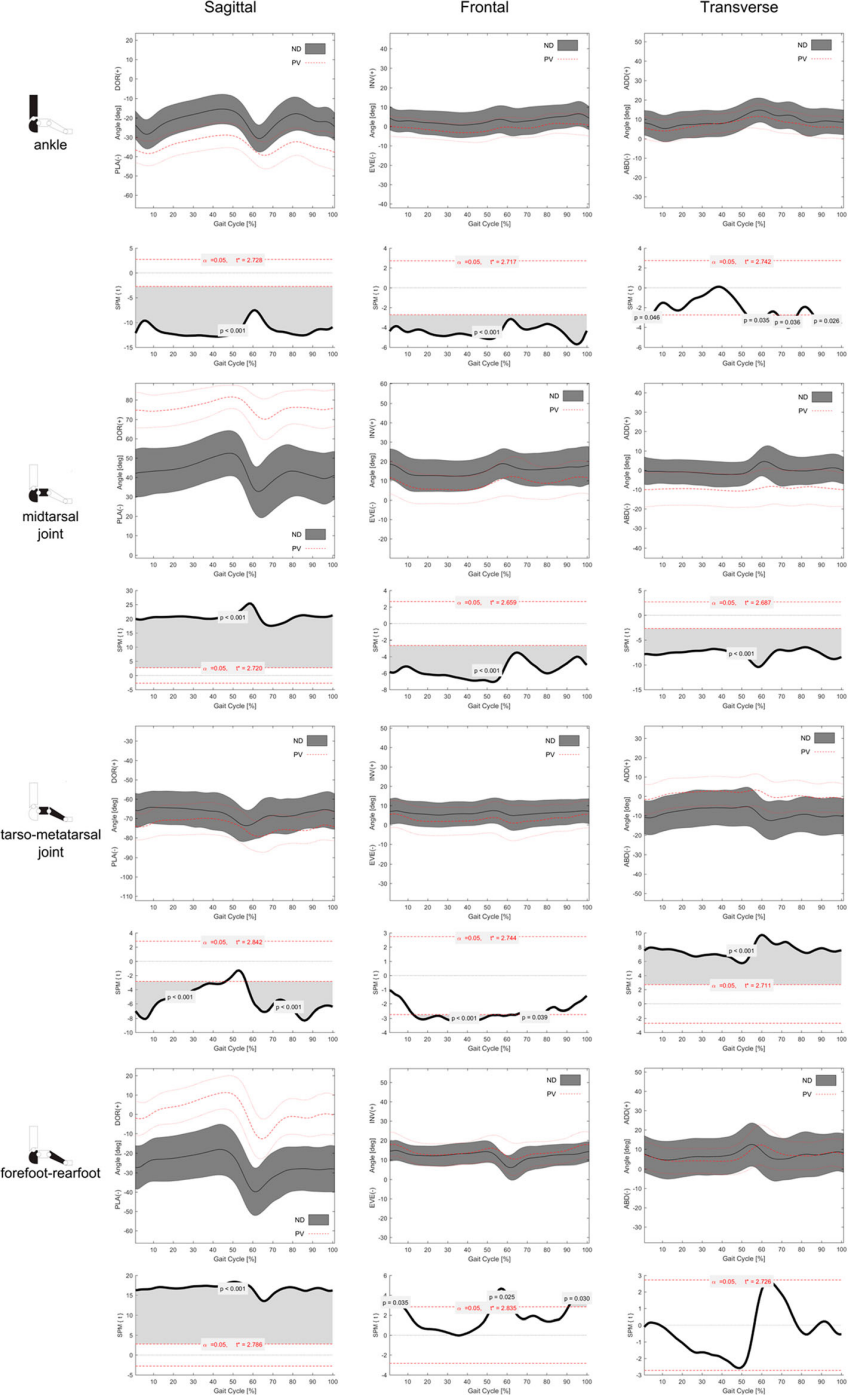
Left to right, sagittal, frontal and transverse plane mean temporal profiles (±1SD) of rotations at, top to bottom, ankle, midtarsal, tarso-metatarsal and forefoot-hindfoot joints in the plano-valgus (PV) and normally-developed feet (ND) groups. Below each plot, statistical parametric mapping has been used to visualize differences in rotation time-histories between PV and ND groups

**Fig. 5:**
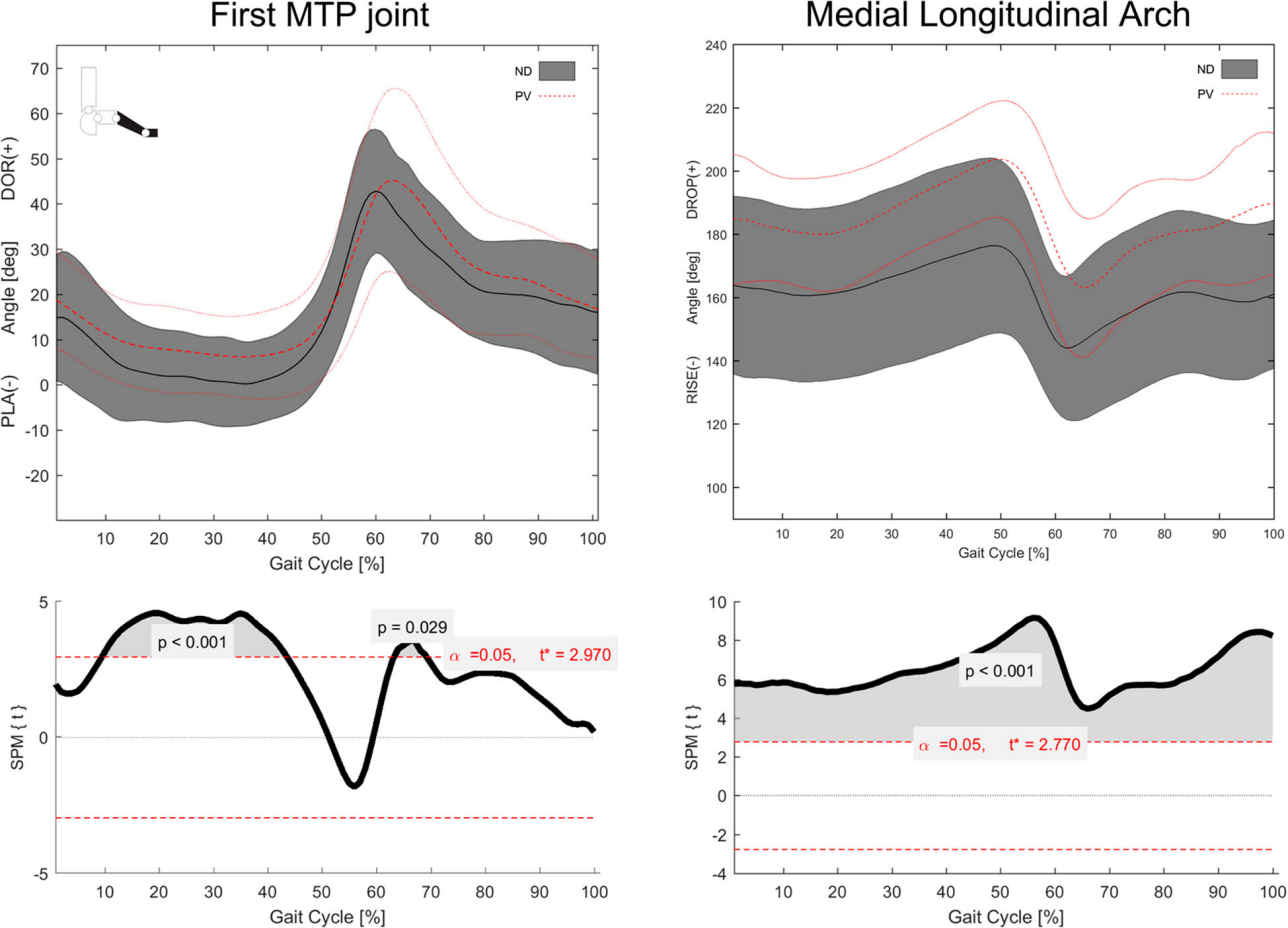
Left**,** mean temporal profile (±1SD) of first metatarsophalangeal joint dorsiflexion/plantarflexion in the plano-valgus (PV) and normally-developed feet (ND) groups. Right, mean temporal profiles (±1SD) of MLA deformation during gait cycle in the PV and ND groups. Bottom, statistical parametric mapping has been used to assess differences in metatarsophalangeal joint rotation and MLA deformation between the two groups

Consistent patterns of foot joint rotations during normalized gait cycle were observed in the two groups (Figs. 4 and 5).

In the PV group, metatarsus and midfoot were more dorsiflexed with respect to the hindfoot than those in the ND group throughout the whole gait cycle. In addition, the midfoot was more everted and abducted with respect to the calcaneus. The calcaneus was more plantarflexed and everted with respect to the shank, and the forefoot was more plantarflexed and more adducted with respect to the midfoot. The forefoot was also more inverted to the calcaneus at push-off (52–62% of gait cycle) and prior-to and in early stance (90–100% and 0–7% of gait cycle).

The first metatarsophalangeal joint was more dorsiflexed throughout most of the stance duration, and showed a larger maximum dorsiflexion at push-off in the PV group (Fig. 4).

The MLA was lower (i.e. larger MLA angles) in the PV group during the whole gait cycle (Fig. 4).

### RoM analysis

The distribution of the joints RoM in the three anatomical planes are reported in Table 4. In the PV group, RoMs in the sagittal and transverse planes were generally lower than those in the ND group, whereas frontal-plane RoMs were larger. However, in accordance with the Bonferroni corrected significance level (α = 0.01), only the sagittal-plane RoM of the midtarsal joint, the frontal- and transverse-plane RoMs of the tarso-metatarsal joint, and the frontal plane RoM between the forefoot and hindfoot were statistically different between the two groups. On average, sagittal- and transverse-the midtarsal joint, the frontaplane RoMs in the PV group were respectively 2.2 to 6.7 degrees, 0.9 to 6.2 degrees smaller than those in the ND group across all foot joints; frontal plane RoMs in the PV group were between 1.3 to 3.6 degrees larger than those in the ND group. MLA deformation was significantly larger in the PV group.

**Table 4:** Sagittal, frontal and transverse plane RoM (deg, median [25 75%)] of ankle, midtarsal, tarso-metatarsal, and forefoot-hindfoot joints during gait cycle in the PV and ND groups

Foot joint	PV			ND			*p*		
	Sagittal	Frontal	Transverse	Sagittal	Frontal	Transverse	Sagittal	Frontal	Transverse
Ankle	14.2 [11.4 17.6]	8.4 [6.9 9.9]	10.7 [8.7 12.5]	17.7 [15.6 19.9]	7.7 [6.4 8.4]	11.4 [10.1 13.4]	0.017	0.028	0.037
Midtarsal	15.9 [12.1 19.2]	11.3 [9.3 13.9]	8.3 [5.8 10.7]	22.2 [19.5 24.9]	9.6 [7.8 11.8]	9.7 [7.9 11.4]	< 0.01	0.026	0.05
Tarso-metatarsal	14.8 [12.3 18.5]	10.4 [7.7 13.1]	8.8 [6.9 10.9]	14.4 [12.0 17.6]	6.8 [5.6 8.1]	11.0 [8.9 13.7]	> 0.05	< 0.001	< 0.01
Forefoot-hindfoot	28.4 [23.0 32.0]	13.9 [10.9 17.5]	11.5 [9.4 17.7]	25.8 [22.9 28.5]	12.3 [10.3 13.9]	13.3 [10.4 16.5]	0.03	0.002	> 0.05
First MTP joint	48.6 [40.0 62.3]	–	–	51.6 [45.1 59.0]	–	–	> 0.05		
MLA	56.0 [45.0 71.0]	–	–	36.0 [31.0 44.0]	–	–	< 0.001		

Sagittal-plane RoM of the first metatarso-phalangeal joint and medial longitudinal arch (MLA) deformation are also reported. Mann-Whitney statistical significant differences in RoM between PV and ND groups are shown on the right (α = 0.01)

## Discussion

The paediatric foot has been extensively studied clinically and radiographically. However, clinical evaluation allows only quantification of the hindfoot frontal-plane alignment in upright standing position. While x-ray imaging can provide accurate information on the two-dimensional alignment of the foot bones, the exposure to ionizing radiation and the complexity of coronal plane measurements strongly limit its use for PV diagnosis. Therefore, skin-markers based gait analysis and multisegment foot protocols have been increasingly used to provide objective evaluation of foot posture and kinematics in the paediatric PV population. Compared to the normal-arched control feet, paediatric PV feet have been shown to present increased hindfoot eversion, and greater forefoot abduction and supination (Table 1). In the lower limb joints, larger external hip rotation in early stance and larger anterior pelvic tilt were observed in 5 years old children with bilateral valgus hindfoot [14].

**Fig. 6:**
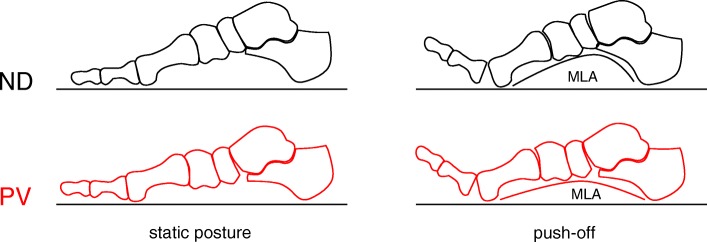
Diagrammatic representation of the sagittal-plane intersegmental orientation in double-leg support static posture (left), and at push-off (right) for the normally-developed (top) and plano-valgus feet (bottom). Calcaneus, midfoot, metatarsus and hallux segments were oriented according to the inter-participant average foot joint rotations recorded in the static acquisition (see Fig. 2) and at push-off (around 60% of the gait cycle; Fig. 3). Please note that the talus could not be tracked by the present foot protocol, and is only shown to improve image interpretation

To the best of the authors’ knowledge, this is the first time a detailed multi-segment postural and kinematic analysis of the paediatric asymptomatic PV has been performed. The present functional evaluation has confirmed that significant alterations are present in PV with respect to age-matched normally developed feet. The differences observed between PV and ND in static posture were consistent with the diagnosis of plano-valgus foot condition, and were strongly related to those observed in gait kinematics. Similar to what was reported in previous studies, the PV hindfoot was significantly everted [13, 16–18], and plantarflexed [16] relative to the tibia. In the PV group, in accordance with [15], MLA deformation was significantly larger and the hallux was more dorsiflexed throughout most of stance duration. This may be related to reduced efficiency of those anatomical structures spanning the midfoot, such as plantar intrinsic foot muscles and aponeurosis, altering the arch rising effect of the windlass mechanism [27] (Fig. 6). This mechanism was shown to occur also in early stance [28], thus alteration of its efficacy may affect foot biomechanics in the loading response phase, when the hindfoot everts and the foot arch lowers to absorb the ground reaction forces. Evidence of kinematic alteration across the medial longitudinal arch in the PV group appears consistent with a recent morphometric analysis of pes-planus in which a reduced cross-sectional area of the 1st ray intrinsic muscles, and reduced plantar fascia thickness were observed [29].

In addition to the current understanding of PV function, it was possible to better identify in which joints most of the static and kinematic alterations that characterize pediatric flexible pes-planus are present. The midtarsal joint was significantly dorsiflexed, everted and abducted throughout gait, and the tarso-metatarsal joint was plantarflexed and slightly adducted. As a result, no significant abduction was observed in the forefoot with respect to the hindfoot. RoM analysis revealed some novel information on the mechanics of the pediatric flexible plano-valgus foot during gait. In the PV group, increased frontal plane RoM was observed at the tarso-metarsal joint and between forefoot and hindfoot, whereas sagittal-plane RoM was reduced at the midtarsal joint. In accordance with the joint posture measured in static conditions, the PV midtarsal joint was more dorsiflexed throughout the whole gait cycle with respect to the ND; however sagittal-plane RoM of the midtarsal joint was reduced. In the frontal plane, the PV group showed increased ankle and midtarsal joint eversion both in terms of offset to the corresponding ND profiles and, albeit not statistically significant, in RoM. From a biomechanical perspective this may be due to the midtarsal joint having reached the largest possible dorsiflexion allowed by skeletal and ligaments constraints (Fig. 6). Conversely, extra mobility is allowed in the frontal plane as the flexible plano-valgus foot can reach a more everted posture compared to normally developed feet.

In accordance with what observed by Lin et al. [4] on a slightly younger PV population, but in contrast with other reports [13, 15], the PV group walked with reduced stride length and walking speed. This is normally associated with some functional deficit, and may be due to the significant foot postural alterations displayed by those children included in the PV subgroup. However, kinetic and pressure analyses could not be performed due to limitations of the present setup, thus functional impairments and compensations in the lower limb joints associated with PV kinematic alterations shall be addressed in future investigations. Although PV kinematics could not be compared to a speed-matched gait data set, this appeared to have only marginally affected the differences in joint kinematics between PV and ND, as most of the kinematic alterations were strongly consistent with those measured in static posture. Moreover, the effect of cadence on foot joints range of motion was found to be significant mainly between slow- and fast-walking cadence, in the frontal and transverse planes only [30]. It should also be highlighted that only 20 PV adolescents were analysed, and that the age was limited to 13 years - the age at which children’s feet reach full skeletal maturity [2, 5, 31] and clinical assessment of pes-planus condition is recommended at the authors’ Institution. These limitations somehow restrict the generalizability of these results to a larger plano-valgus paediatric population. Nevertheless this study provides objective information on foot kinematics for a critical paediatric population presenting significant alterations of foot skeletal morphology, and for which the choice of the most appropriate treatment is still highly debated. In terms of limitations of the methodology, while the Rizzoli Foot Model is a widely used and extensively validated kinematic protocol [19, 20, 32] presenting a balance of moderate repeatability and reasonable test-retest error [33], it should be reminded that kinematic analysis of foot joints via skin-markers is intrinsically affected by skin-motion artifacts. The magnitude of this error on the calculated foot joints RoM is difficult to determine with precision, thus making the interpretation of the absolute joint rotations rather difficult. However, this has likely biased both groups to a similar extent, with negligible effects on the differences detected between the groups.

## Conclusions

Multisegment kinematic analysis based on skin-markers has proved to be an effective non-invasive technique to detect functional alterations of foot segments in the three anatomical planes, which can not be identified clinically or radiographically. According to the results of this study, significant postural and kinematic alterations are present at the midtarsal and tarso-metarsal joints of adolescents with asymptomatic PV with respect to normal-arched healthy feet. While larger dorsiflexion of midfoot joints and greater MLA collapse provide evidence for a hindered windlass mechanism, further studies should nonetheless be sought to fully comprehend the effect of these alterations to PV function, as well as modifications following surgical correction by different techniques.

## Abbreviations

MLA: Medial longitudinal arch
ND: Normally-developed foot
PV: Plano-valgus foot
RoM: Range of motion
